# Chronic Consumption of Bovine Dairy Milk Attenuates Dietary Saturated Fatty Acid-Induced Blood-Brain Barrier Dysfunction

**DOI:** 10.3389/fnut.2020.00058

**Published:** 2020-05-06

**Authors:** Zachary D'Alonzo, Virginie Lam, Michael Nesbit, Liam Graneri, Ryu Takechi, John C. L. Mamo

**Affiliations:** ^1^Curtin Health Innovation Research Institute, Faculty of Health Sciences, Curtin University, Perth, WA, Australia; ^2^School of Pharmacy and Biomedical Sciences, Faculty of Health Sciences, Curtin University, Perth, WA, Australia; ^3^School of Publich Health, Faculty of Health Sciences, Curtin University, Perth, WA, Australia

**Keywords:** bovine dairy milk, saturated fatty acids, blood-brain barrier, neuroinflammation, cytokines

## Abstract

Ingestion of Western-diets enriched in long chain saturated fatty acids (LCSFA) are associated with increased risk of blood-brain barrier (BBB) dysfunction and neurovascular inflammation. Potential mechanisms include vascular insult as a consequence of metabolic aberrations, or changes in capillary permeability resulting in brain parenchymal extravasation of pro-inflammatory molecules. Bovine dairy milk (BDM) is potentially a significant source of dietary LCSFA, however, BDM contains an array of bioactive molecules purported to have vascular anti-inflammatory properties. This study investigated the effects of full cream (4% total fat) and delipidated (skim) BDM on BBB integrity and neuroinflammation in wild-type mice. Mice consuming substantial amounts of full cream or skim BDM with LCSFA-enriched chow were dyslipidemic compared to control mice provided with standard chow and water. However, there was no evidence of BBB dysfunction or neuroinflammation indicated by parenchymal abundance of immunoglobulin G and microglial recruitment, respectively. Positive control mice maintained on an LCSFA-enriched diet derived from cocoa-butter and water, had marked BBB dysfunction, however, co-provision of both full cream and skim milk solutions effectively attenuated LCSFA-induced BBB dysfunction. In mice provided with low-fat chow and full cream BDM drinking solutions, there were substantial favorable changes in the concentration of plasma anti-inflammatory cytokines. This study suggests that consumption of BDM may confer potent vascular benefits through the neuroprotective properties exuded by the milk-fat globule membrane moiety of BDM.

## Introduction

Risk factors for vascular dysfunction such as dyslipidaemia and obesity are associated with early onset and progression of neurodegenerative disorders such as Alzheimer's disease and vascular dementia (1). Chronic ingestion of Western-styled diets enriched in long chain saturated fatty acids (LCSFA) exacerbate metabolic parameters that results in a chronic state of inflammation which can lead to vascular dysfunction (2–4). The cerebral micro-vasculature is particularly sensitive to pro-inflammatory insults, realized as increased capillary permeability and potential extravasation of toxic molecules within brain parenchyme. Exaggerated intake of dietary LCSFA has been shown to increase blood-brain barrier (BBB) permeability by reducing expression of tight-junction proteins in cerebral endothelium (5). The latter is thought to occur as a consequence of exaggerated endothelial exposure to circulating pro-inflammatory cytokines (6, 7). In genetically un-manipulated mice maintained on a modestly LCSFA-enriched diet, plasma-derived proteins and macromolecules were indicated within brain parenchyme, triggering microglial and astrocyte activation, exacerbating neurovascular inflammation (8).

Dietary LCSFA's are commonly found in animal products such as meat and dairy, staple food commodities in Western styled diets. Standard bovine dairy milk (BDM) preparations contain ~4% w/w fat, of which 70% is principally palmitic and stearic fatty acids (9, 10). However, unlike processed meat products rich in LCSFA, BDM also contains bioactive components in the lipid fraction with potent anti-inflammatory actions. Several studies have analyzed the anti-inflammatory effects of various BDM-derived molecules including protein isolates, fractions of bio-active lipids and indeed, calcium. The milk fat globule membrane (MFGM) which chaperones and encases BDM lipids, contains an estimated 120 proteins, some of which have been demonstrated to potently modulate immune function (11–13). In one study, lactadherin, a protein naturally enriched in BDM, markedly suppressed inflammation in mice induced with a fluid percussion injury (12). Similarly, mucins have been shown to play a role in the innate immune system and may attenuate bacterial infections (13).

Bovine dairy milk is rich in phospholipids which have been reported to suppress inflammatory reactions. Bovine dairy milk phospholipids reduce endoplasmic reticulum stress in neuronal cells, implicated in development of neurodegenerative disorders (14). Moreover, relevant to brain function, milk phospholipids such as phosphatidylcholine and sphingomyelin, were found to promote cell structural integrity (15), increase neurovascular density (16) and promote cognitive development and function (16, 17).

Calcium enriched foods such as BDM may confer vascular benefits indirectly, by positively modulating metabolism, obesity and lipid homeostasis. Bruckbauer et al. (18) suggested that dietary calcium may reduce the level of calcitriol in the blood, which regulates adipocyte reactive oxygen species (ROS). Consistent with the latter, mice supplemented with dietary calcium intake had reduced levels of NADPH oxidase and a reduction in ROS production, both of which are associated with inflammation.

Dairy milk contains compounds and macromolecules which potentially promote, or inhibit vascular inflammation, however the synergistic net effect of BDM on cerebral capillary integrity has not been previously reported. This study explored putative effects of alternate BDM formulations on indices of the BBB and neurovascular inflammation in wild-type mice. Moreover, potential synergistic effects with a pro-atherogenic LCSFA enriched chow diet was also considered.

## Materials and Methods

### Animals and Dietary Interventions

**Figure 1:**
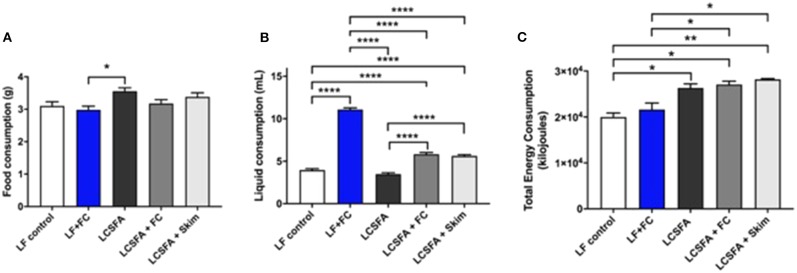
Food, liquid and total energy consumption **(A)** Food consumption was measured weekly and average daily consumption was calculated per mouse for each of the LF, LCSFA, LF+FC, LCSFA+FC, and LCSFA+Skim groups **(B)** Liquid consumption was measured daily and averaged per mouse for each of the LF, LCSFA, LF+FC, LCSFA+FC, and LCSFA+Skim groups. **(C)** Total cumulative energy consumption was calculated at the end of the 13-week intervention per group. All data were calculated and expressed as mean±SEM. Asterisks indicate statistical significance *P < 0.05; **P < 0.01; ****P < 0.0001; non-parametric multiple comparison; *n* = 10.

Male 5-week old C57BL/6J mice were purchased from the Animal Resources Center (WA, Australia). Mice were randomly allocated into 5 experimental groups (*n* = 10). Low-fat control group was fed standard low-fat maintenance chow containing 4% (w/w) fat as monounsaturates (AIN 93M, Specialty Feeds, WA, Australia). The high LCSFA positive control group was maintained on a semi-synthetic diet containing 40% of digestible energy derived from cocoa butter (23% (w/w), SF07, Specialty Feeds, WA, Australia). Two other groups were allocated a 20% full cream (FC) milk solution diluted with water, with one group receiving high LCSFA diet (LCSFA + FC) and the other receiving low-fat control chow (LF + FC). The final group was 20% skim milk with a high LCSFA diet (LCSFA + Skim). Each group was sacrificed at 13 weeks from the start of the dietary intervention. The mice were held in Curtin University Animal Facility with controlled air temperature (22°C), air pressure and a 12-h light/dark cycle. All mice had *ad libitum* access to food and liquid. Milk solutions were replaced daily to prevent rancidity. Liquid consumption was monitored and recorded daily and food consumption was measured weekly for each group (Figure 1). The total amount of energy consumed was calculated by converting all measurements to calories based on the food composition data for each semi-purified diet / liquid to compare between each experimental group (Figure 1C). This study was carried out in strict accordance with the Australian National Health and Medical Council Guidelines and approved by the Curtin University Animal Ethics Committee under project number 2018-03.

### Sample Collection

Following the 13-week intervention, mice were anesthetized with isoflurane gas and blood was collected via cardiac puncture. Mice were killed by exsanguination before brains were removed, washed in chilled PBS, followed by an immersion-fix in 4% paraformaldehyde for 24 h. The tissues were then cryoprotected in 20% sucrose for 72 h at 4°C before being frozen in isopentane/dry ice and stored at −80°C. Blood was left for 30 min at room temperature before serum separation through centrifugation at 4°C at 4, 000 rpm for 10 min. Serum samples were separated in 100 μL aliquots and frozen at −80°C until further analysis.

### 3-Dimensional Assessment of Blood-Brain Barrier Integrity and Neuroinflammation

Blood-brain barrier integrity was analyzed by 3-dimensional semi-quantitative immunomicroscopy detection of cerebral parenchymal immunoglobulin G (IgG) extravasation, as established previously (2, 4, 8). Briefly, 20 μm coronal cryosections of the cerebral right hemisphere were collected on polysine-coated microscope slides. Non-specific binding sites were blocked with 10% goat serum in PBS for 30 min. Sections were then incubated with goat anti-mouse IgG conjugated with Alexa 488 fluorophore (1:200, Invitrogen, USA) at 4°C for 20 h. Sections were then washed with PBS before application of 4′,6-diamidino-2-phenylindole (DAPI) nuclei counterstaining (Invitrogen, USA). Sections were finally mounted with antifade mounting medium. 3-D immunofluorescent images were captured with a spinning disc confocal microscope with a 20 × objective and Volocity imaging software (Version 5.4.2, UltraView Vox, Perkin-Elmer, MA, USA). Each 3-D image consisted of 20 × 2-D images with a 1 μm z-axis distance (1,000 × 1,000 pixels, 346 × 346 μm). An average of 15 images were taken of the cerebral cortex, per mouse. Voxel intensity of diffusing IgG surrounding the periphery of cerebral capillaries was determined for each 3-D image with Volocity 5.4.2 image analysis software by a blinded investigator (Version 5.4.2, UltraView Vox, Perkin-Elmer, MA, USA). The mean voxel intensity was calculated for each mouse and then per group.

The expression of ionized calcium-binding adaptor protein-1 (Iba-1) was determined as a measure of neuroinflammation by indicating microglial recruitment, as described previously (19). Twenty Micrometer thick cryosections of the right brain hemispheres were incubated in Tris-EDTA (T.E) buffer (10 mM, pH 6.0) for 20 h at 37°C for antigen retrieval. Non-specific binding sites were then blocked by 10% goat serum for 30 min, followed by a 20 h incubation with rabbit anti-mouse Iba-1 antibody (Wako Chemicals, Japan) at a concentration of 1:500. Brain sections were then incubated with goat anti-rabbit IgG conjugated with Alexa 488 fluorophore at a concentration of 1: 500 for 2 h at room temperature (Invitrogen, USA). Nuclei were then counterstained with DAPI.

An average of 15 × 3-D images were captured to represent the cerebral cortex. The voxel intensity of Iba-1 staining was determined by using Volocity 5.4.2 image analysis software (Version 5.4.2, UltraView Vox, Perkin-Elmer, MA, USA).

### Serum Triglycerides and Cholesterol Measurements

Serum triglycerides and total cholesterol concentrations were determined by using colorimetric assays according to the manufacturer's instructions (Randox, UK). Briefly, 2 μL of serum was added to 200 μL of reagent and incubated for 10 min at room temperature (RT). Optical density was measured using an EnSight^TM^ multimode plate reader (Perkin Elmer, USA) at a wavelength of 546 nm. Assay standards were used to develop a linear regression line in which sample lipid concentrations were interpolated from.

### Serum Insulin Analysis

Serum levels of insulin were measured using an Ultrasensitive Mouse Insulin ELISA kit (Mercodia, Sweden), as per the manufacturer's instructions. Briefly, 25 μL of serum was added to 100 μL of enzyme conjugate and incubated for 2 h on a plate shaker. Samples were washed and then combined with TMB substrate for 15 min at RT. Fifty Microliter of stop solution was added to each well and samples were then read on an EnSight^TM^ multimode plate reader (Perkin Elmer, USA) at a wavelength of 450 nm. Assay standards were used to develop a linear regression line in which sample concentrations were interpolated from.

### Serum Cytokine Beads Array Analysis

Detection of cytokines was carried out using the commercially available BD cytometric bead array (CBA) Mouse Th1/TH2/TH17 cytokine kit (BD, San Jose, CA, USA) as established previously with some minor adaptions (2). In brief, 10 μL of serum sample was used in 96 well plates and reagent, containing mouse cytokine capture beads with distinct fluorescent intensities, was proportionally adjusted to suit sample size. Samples were measured using a BD Fortessa Flow Cytometer (BD, San Jose, CA, USA) and all sample analysis and data were collected using FlowJo (FlowJo, OR, USA) flow cytometry software.

### Statistical Analysis

All data were calculated and expressed as mean ± SEM. In order to assess the effects of LCSFA diet and dairy milk consumption on cerebrocapillary BBB, neuroinflammation and peripheral cytokine profile, all normally distributed data were analyzed with one-way analysis of variance (ANOVA) followed by Fisher's least significant difference (LSD) *post hoc* test for multiple comparison (GraphPad Prism 8, USA). All data that were not normally distributed were analyzed by Krusal-Wallis test with Dunn's *post hoc* test (GraphPad Prism 8, USA). Statistical significance was detected at *p* < 0.05 (*n* = 10).

## Results

### Dietary Intake of BDM and Chow Consumption Patterns

This study investigated the putative effects of chronic ingestion of full cream or skim BDM on neurovascular integrity and neuroinflammation in genetically unmanipulated wild-type mice. The milk formulations were diluted to either 20% of commodity strength and provided with standard low-fat chow, or to mice maintained on a pro-atherogenic LCSFA- enriched diet. The milk drinking solutions were well-tolerated and no adverse events were reported for the 12-week treatment period. The ingestion of milk solutions compared to treatment groups provided with water is indicated in Figure 1B. Low-fat fed mice randomized to full cream milk had more than a doubling of intake of liquid ingestion. LCSFA-fed mice provided with full cream and skim milk had comparable and modest increased intake of drinking solutions. Provision of the LCSFA diet alone had no significant effect on water intake compared to mice on a low-fat diet, but a significant increase in the intake of both full cream and skim solutions was seen in the LCSFA + FC and LCSFA + Skim groups.

Average chow consumption and the total energy intake for treatment groups (Figures 1A,C, respectively) demonstrated that *ad libitum* provision of LF + FC did not increase net energy intake. Conversely, LCSFA-fed mice provided with skim milk had ~20% greater energy intake compared to the low-fat controls for the duration of the study. Mice provided with diets enriched in LCSFA had greater total energy intake, principally as a consequence of the higher caloric content of the chow formulation.

### Serum Lipids and Insulin Profile

**Figure 2:**
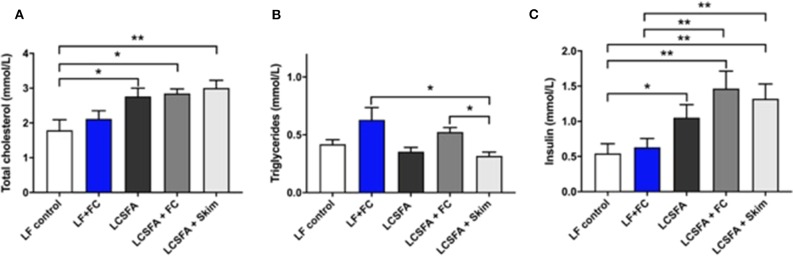
Blood lipid profile for each of the dietary groups, LF, LCSFA, LF+FC, LCSFA+FC, and LCSFA+Skim **(A)** Total cholesterol, **(B)** Serum triglycerides, and **(C)** Serum insulin. All data were calculated and expressed as mean±SEM. Asterisks indicate statistical significance *P < 0.05; **P < 0.01; one-way ANOVA followed by Fisher's least significant difference (LSD) *post hoc* test; *n* = 9–10.

Treatment effects on serum lipids and insulin sensitivity are depicted in Figure 2. Similar to mice fed a LCSFA-enriched diet, groups maintained on both full cream and skim BDM formulations with LCSFA-enriched chow, demonstrated hypercholesterolemia (Figure 2A). Mice consuming substantial quantities of full cream milk with low-fat chow or LCSFA-enriched chow, had a modest increase in serum triglycerides (Figure 2B). In LCSFA + Skim fed mice, where ingestion volumes were less exaggerated, serum triglycerides were not significantly different from low-fat fed controls, however, there was evidence of heightened serum cholesterol. This study confirms previous studies that mice maintained on LCSFA enriched diets were normotriglyceridemic, but had exaggerated serum cholesterol. Treatment groups with exaggerated serum cholesterol (LCSFA; LCSFA + FC; LCSFA + Skim), had increased levels of serum insulin compared to age-matched low-fat fed controls (Figure 2C).

### Cerebrovascular Permeability and Neuroinflammation

**Figure 3:**
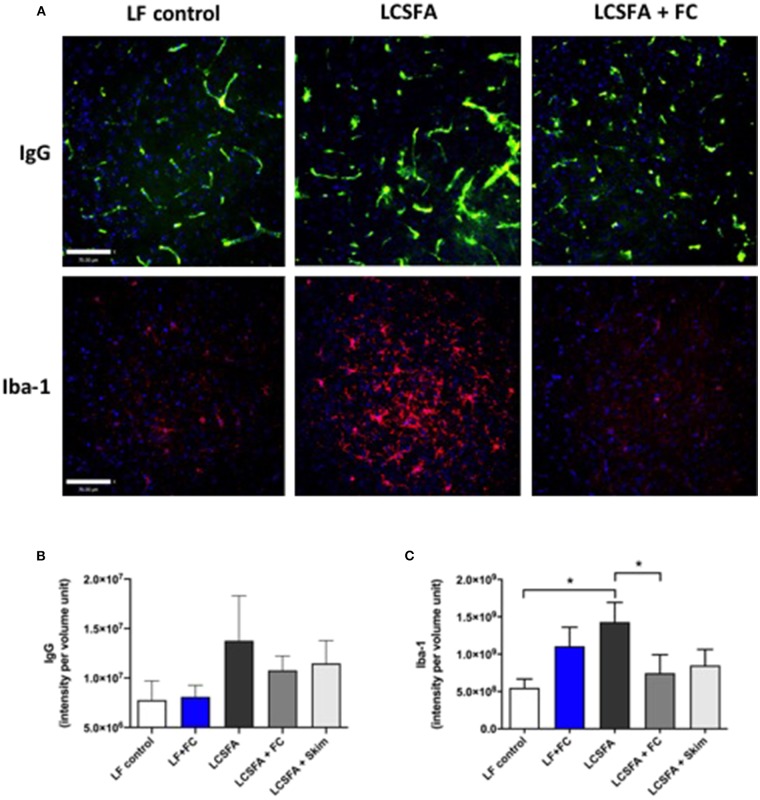
Semi-quantitative immunofluorescent measures of cerebrovascular integrity and neuroinflammation **(A)** Representative confocal immunomicrographs of the cerebral cortex that display IgG staining of vessels and associated extravasation in green (top frames) and Iba1 staining microglia indicated in red (bottom frames), in mice maintained on LF, LCSFA, and LCSFA+FC. Scale bars indicate 70 μm **(B)** BBB permeability was determined by semi-quantitative 3-dimensional fluorescent immunomicroscopy analysis of IgG extravasation in the cerebral cortex **(C)**. Neuroinflammation was measured by semi-quantitative confocal microscopy analysis of Iba1 expression in the brain parenchyme of the 5 intervention groups. All data were calculated and expressed as mean±SEM. Asterisks indicate statistical significance *P < 0.05; non-parametric Kruskal-Wallis test, followed by Dunn's multiple comparisons test; *n* = 10.

Brain parenchymal IgG and Iba-1 were used as surrogate markers of capillary permeability and neurovascular inflammation, respectively (Figure 3A). This study found that despite a heightened triglyceride response induced by the provision and substantial ingestion of bovine dairy milk formulations, mice randomized to low-fat chow with *ad libitum* access to full cream milk had comparable parenchymal IgG abundance to the LF group (Figure 3B). Additionally, both groups of LCSFA-fed mice maintained on either full cream or skim BDM formulations, showed lower capillary permeability indices when compared to the LCSFA-fed positive control group.

Extravasation of plasma-IgG to brain parenchyme was associated with microglial activation, determined as Iba-1 (Figure 3C). Cerebral Iba-1 abundance was significantly greater in LCSFA-fed positive control mice, but not markedly elevated in mice randomized to groups consuming any of the BDM formulations. Remarkably, LCSFA-fed groups maintained on the full cream and skim BDM, demonstrated substantive attenuation of Iba-1 expression.

### Peripheral Cytokine Analysis

**Figure 4:**
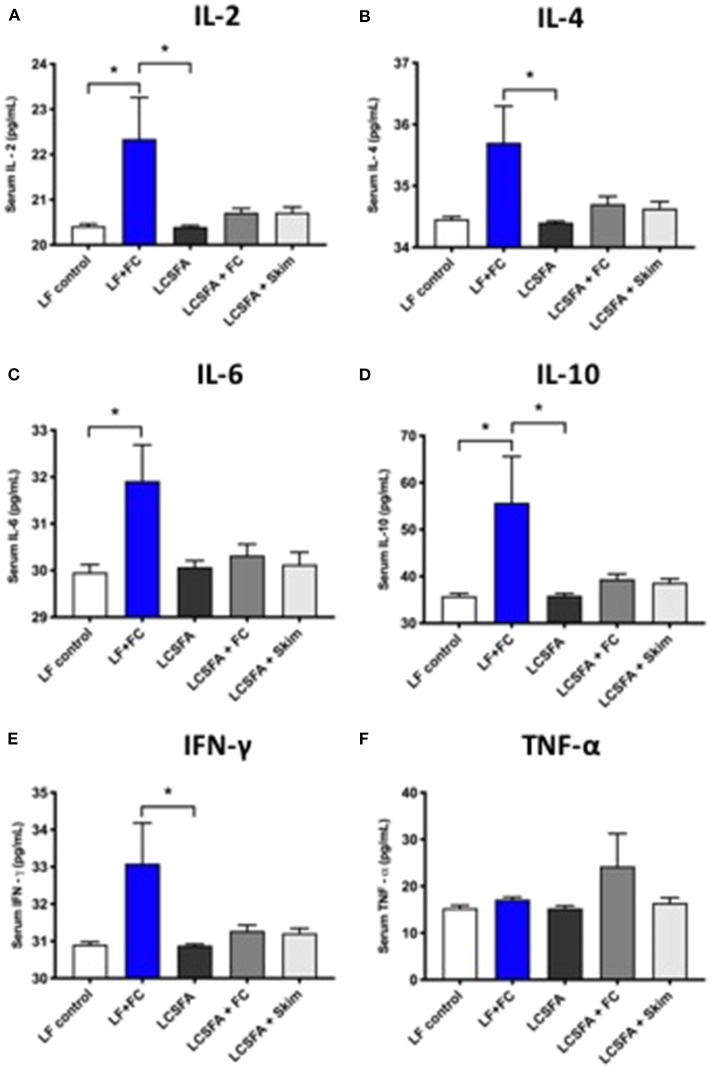
Serum inflammatory cytokine panel **(A)** IL-2, **(B)** IL-4, **(C)** IL-6, **(D)** IL-10, **(E)** IFN-⋎, and **(F)** TNF-α, and were analyzed by flow cytometry for each of the groups LF, LCSFA, LF+FC, LCSFA+FC, and LCSFA+Skim. All data were calculated and expressed as mean±SEM. Asterisks indicate statistical significance *P < 0.05; non-parametric Kruskal-Wallis test, followed by Dunn's multiple comparisons test; *n* = 9–10.

Capillary function may be modulated by exaggerated vascular exposure to pro- and anti-inflammatory cytokines. This study reports that the provision of full cream BDM in conjunction with a control diet, markedly increased serum concentrations of IL-2, IL-4, IL-6, IL-10, and IFN-γ, irrespective of chow treatment (Figure 4). Serum TNF-α was substantially elevated in LCSFA-fed mice maintained on full cream BDM (Figure 4F). Interestingly. LCSFA fed mice provided water had no remarkable change in their cytokine profile.

## Discussion

It has previously been described that a diet rich in LCSFA can induce BBB dysfunction (3). Full cream BDM is rich in LCSFA's, however, it also contains many other bioactive components that may confer neuro-protective benefits. Whilst multiple studies have broadly investigated the physiological effects of dairy products, none have explored the direct impact of BDM on the integrity of the BBB. This study examined, for the first time, the putative effects of full cream or skim BDM on neurovascular integrity and metabolic factors that may otherwise contribute to cerebrovascular dysfunction.

Wild-type mice provided milk interventions drank markedly more than mice provided water. This behavioral difference in drinking was independent of the chow formulation provided (control chow, or LCSFA-enriched chow) and was sufficient to induce modest changes in their cholesterol and triglyceride response. Despite comparable levels of hypercholesterolemia in BDM-fed groups maintained on LCSFA chow and LCSFA-only mice, and pronounced triglyceride concentrations in full-cream BDM groups, the changes in lipid indices were not associated with cerebral capillary dysfunction or neurovascular inflammation. The attenuation of capillary dysfunction and marked attenuation of neuroinflammation occurred concomitant with increased levels of anti-inflammatory cytokines in mice maintained on the LF+FC formulation. However, low-fat and skim milk interventions were found to reduce inflammation without significant effects on anti-inflammatory cytokine abundance. The latter suggests that BDM may also regulate neurovascular function and inflammation through other mechanisms, including perhaps via direct modulation of the capillary endothelium. Milk formulations were provided in 20% dilutions and would be physiologically relevant if intake aligned with that of the control mice. However, intake of milk formulations were significantly greater. Liquid consumption was the highest among the LF + FC group, followed by LCSFA + FC and LCSFA + Skim. Mice provided milk solutions concomitant with LCSFA-enriched chow showed modestly higher ingestion of drinking solutions compared to the LCSFA and water, but markedly less than the LF + FC group. Treatment effects on satiety signaling may underlie differences observed in chow and supplementary liquid (milk formulations/water) intake. Individual consumption of liquid, chow and energy intake could not be deliberated due to grouped housing/diet conditions. The consumption rates of both chow and liquid depict the cumulative energy consumption, in that the LCSFA-chow receiving groups had the greatest overall energy intake.

Groups maintained on full cream BDM formulations (LF + FC; LCSFA + FC) developed an increased dyslipidemic response that was dose dependent as indicated by serum triglyceride levels. The liquid consumption data (Figure 2B) suggests an association of plasma triglyceride homeostasis relative to the ingestion specifically of BDM-fat. The LCSFA + FC and LCSFA + Skim groups had similar ingestion volumes of drink formulations, however, the latter had a significantly lower concentration of blood triglyceride. The observation suggests that the availability of triglycerides. Through digestion and absorption of milk lipids is causally associated with exaggerated hypertriglyceridemia. The triglyceride response may be driving lipogenesis in the full cream BDM groups as a result of an increased availability of triglycerides through digestion and absorption of the milk lipids. No increase in triglycerides were reported in the skim BDM group and may be attributed to the delipidation process. Furthermore, the skim BDM group demonstrated elevated cholesterol and insulin levels that were also substantiated in the full cream fed LCSFA groups. This suggests that within this group, insulin may be driving lipogenesis through a hypersecretion of triglycerides from the liver (20). Elevated serum insulin would also increase expression of lipoprotein lipase, leading to the storage of triglycerides in adipose tissue and also increasing circulating levels of cholesterol (21). Whilst body composition data was not acquired in this study, based on the body weight data collected (not shown) and lipid profiles indicated in Figure 1, obesity may contribute to systemic inflammation through a range of metabolic influences including production of adipocyte ROS and adipocytokines (7, 18, 22).

Despite the notable effect of BDM on an abnormal lipid and insulin profile, immunomicroscopic analysis revealed there was no evidence of BBB leakage, characterized by extravasation of IgG from blood to brain parenchyme, within these groups. More intriguingly, both LCSFA-fed full cream and skim BDM groups revealed modest reductions in BBB indices and indeed indicative that BDM may attenuate dietary LCSFA-induced BBB dysfunction. However, IgG has a molecular weight of 166 kDa, thus the possibility of more subtle changes in BBB permeability cannot be excluded. Although extravasation of smaller biomarker molecules may not be detected, subtle changes in BBB permeability can be indirectly detected through Iba-1 expression, a biomarker of microglial recruitment, of which extravasation is normally associated with. Interestingly, Iba-1 expression is consistently lower for all BDM-maintained groups compared to the LCSFA positive control. Iba-1 is expressed in microglia during their activation by infection or damage to cells, indicating the correlation of chronic consumption of LCSFA with an inflamed state, as previously published (23).

Although LCSFA is known to induce BBB dysfunction, this study suggests that if consumed in conjunction with full cream BDM, both BBB dysfunction and neuroinflammation are modestly attenuated. This provides evidence that BDM may exude neuroprotective effects on BBB integrity. Furthermore, the skim BDM group also showed a decrease in both IgG extravasation and neuroinflammation, suggesting that neuroprotective components could potentially be within the non-lipid moiety of milk. The possibility of certain lipids having neuroprotective effects on the BBB cannot be excluded, however, the skim milk phase reportedly contains a small percentage of phospholipids (24).

As discussed earlier, an increase in pro-inflammatory cytokines can ultimately cause a breakdown of endothelial cell structure. A diet rich in LCSFA can lead to endothelial dysfunction through dylipidemia and obesity effects and is likely to be contributing to the BBB dysfunction observed in the LCSFA control group (6, 7, 18, 22). Mice maintained on control chow and full cream BDM solution had significantly higher levels of cytokines IL-2, IL-4, IL-6, and IL-10, all of which are known to confer anti-inflammatory actions. IL-2 may be anti-inflammatory by suppressing T helper 17, a pro-inflammatory cell type (25). IL-4 has a role in suppressing IL-1 and IL-8, both of which contribute to an inflammatory state (26). IL-6 may function as an anti-inflammatory cytokine through its suppressive effects on TNF-α, but also through its effect on upregulating IL-10 production (27). IL-10 is known to inhibit the production of pro-inflammatory cytokine TNF-α and also ROS, which would otherwise trigger inflammation (25). In a previous study, mice maintained on a BDM-derived diet showed suppression of pro-inflammatory cytokine, TNF-α, concomitant with endothelial dysfunction (28). In the present study, systemic TNF-α was indeed comparable between all BDM-intervened groups and the LF fed control mice with exception to the mice maintained on LCSFA + full cream BDM. The results from this study do not support the direct effects of TNF-α on BBB dysfunction or increased recruitment of microglial cells. In fact, microglial expression was somewhat decreased in the LCSFA + full cream BDM group when compared to the positive control LCSFA and other BDM-fed groups. This may be due to the elevated anti-inflammatory cytokines such as IL-10 having a negating effect on the pro-inflammatory cytokine cascade.

An increase in anti-inflammatory cytokines and protective effect of BDM on the BBB may be the product of phospholipids that are contained within the MFGM. A study by Nagai (14) identified that a mixture of BDM phospholipids may attenuate endoplasmic reticulum stress in neurons that is associated with neurodegenerative diseases, demonstrating the anti-inflammatory role of BDM derived phospholipids. Another study demonstrated that treatment of arthritis in rats with a BDM derived phospholipid, phosphatidylcholine, exhibited anti-inflammatory properties and reduced arthritis even more so than a commercial arthritis medication (29). Collectively, these potent phospholipids may induce an anti-inflammatory effect following chronic consumption of BDM, however, it is more likely that these phospholipids confer protection in an additive context in attenuating the indices cerebrovascular function and neuroinflammation.

BDM contains a significant amount protein as part of its macronutrient profile, with ~20% of the total contributed by whey protein. BDM is also a rich source of calcium and riboflavin, notably in the whey protein fraction, which has proven antioxidant and inflammatory effects, principally through its effects on glutathione activity. Recent studies have implicated the association between cerebral glutathione concentration and consumption of BDM-enriched foods. Dairy products purportedly act as exogenous substrates for glutathione synthesis in the brain and whilst glutathione status was not assessed in this study, it is reasonable to infer that increased consumption of BDM may have contributed to vasculo-protection and attenuation of glial activation, mediated by cerebral antioxidant pathways (30).

Furthermore, whey protein is known to have anti-inflammatory effects, with studies indicating significant reductions in C-reactive protein as a response to whey supplementation (31). It is unlikely that the increased levels of anti-inflammatory cytokines are a result of MGFM-derived bioactive proteins from BDM, as there is no previous evidence that MFGM derived proteins may reduce inflammation directly. However, certain intrinsic components of the MFGM such as mucin and lactadherin, have been shown to have neuroprotective properties and indeed, reports have implicated the role of lactadherin in supressing inflammation and apoptosis and more importantly, promote angiogenesis (32–34). Some mucin proteins may indirectly prevent a systemic influx of pro-inflammatory cytokines due to its ability to direct macrophage phagocytosis of disease causing bacteria that can induce an inflammatory response (13). In mucin 1-knockout mice infected with *Streptococcus pneumoniae*, the ability to phagocytose and clear the bacteria was suppressed compared to wild-type mice, indicating a protective role of mucin 1 (13). Lactadherin has been shown to help phagocytose cell debris in the brain, and also reduce cerebral edema, which may be associated with inflammation, following physical trauma (11, 12). Collectively, these factors strongly suggest that the protein moieties of the BDM-MFGM may confer neuroprotective mechanisms to attenuate LCSFA-induced capillary dysfunction and neuroinflammation and indeed, warrants further investigation.

Previously, a diet high in LCSFA has been described in inducing BBB dysfunction, though the direct mechanism is unknown. The present study provides evidence that BDM may attenuate dietary LCSFA induced BBB dysfunction and neuroinflammation through the bioactive protein moieties of BDM. Furthermore, evidence of BDM induced dyslipidemia had no effect on BBB dysfunction or neuroinflammation, most likely due to the protective effects of anti-inflammatory cytokines, or by an increase in other BDM bioactive compounds such as phospholipids, calcium and lactadherin. Collectively, the findings in this study further supports the neuroprotective value of BDM-derived compounds and may provide insight into future preventative treatments to help reduce the risk of vascular-related neurodegenerative diseases.

## Data Availability Statement

All datasets generated for this study are included in the article.

## Ethics Statement

The animal study was reviewed and approved by Curtin University Animal Research Ethics Committee.

## Author Contributions

ZD'A, VL, MN, and LG were involved in data collection, data analysis, and manuscript preparation. ZD'A, VL, RT, and JM were involved in study design, results interpretation, data analysis, preparation, and writing of manuscript.

## Conflict of Interest

The authors declare that the research was conducted in the absence of any commercial or financial relationships that could be construed as a potential conflict of interest.
